# CLEC14a-HSP70-1A interaction regulates HSP70-1A-induced angiogenesis

**DOI:** 10.1038/s41598-017-11118-y

**Published:** 2017-09-06

**Authors:** Jihye Jang, Mi Ra Kim, Taek-Keun Kim, Woo Ran Lee, Jong Heon Kim, Kyun Heo, Sukmook Lee

**Affiliations:** 1 Research Center, Scripps Korea Antibody Institute, Chuncheon, South Korea; 2 Department of System Cancer Science, Graduate School of Cancer Science and Policy, National Cancer Center, Goyang, South Korea; 3 Cancer Cell and Molecular Biology Branch, Research Institute, National Cancer Center, Goyang, South Korea; 4 New Experimental Therapeutics Branch, Research Institute, National Cancer Center, Goyang, South Korea

## Abstract

CLEC14a (C-type lectin domain family 14 member) is a tumor endothelial cell marker protein that is known to play an important role in tumor angiogenesis, but the basic molecular mechanisms underlying this function have not yet been clearly elucidated. In this study, using various proteomic tools, we isolated a 70-kDa protein that interacts with the C-type lectin-like domain of CLEC14a (CLEC14a-CTLD) and identified it as heat shock protein 70-1A (HSP70-1A). Co-immunoprecipitation showed that HSP70-1A and CLEC14a interact on endothelial cells. *In vitro* binding analyses identified that HSP70-1A specifically associates with the region between amino acids 43 and 69 of CLEC14a-CTLD. Competitive blocking experiments indicated that this interacting region of CLEC14a-CTLD significantly inhibits HSP70-1A-induced extracellular signal-regulated kinase (ERK) phosphorylation and endothelial tube formation by directly inhibiting CLEC14a-CTLD-mediated endothelial cell-cell contacts. Our data suggest that the specific interaction of HSP70-1A with CLEC14a may play a critical role in HSP70-1A-induced angiogenesis and that the HSP70-1A-interacting region of CLEC14a-CTLD may be a useful tool for inhibiting HSP70-1A-induced angiogenesis.

## Introduction

Angiogenesis is a physiological process through which new blood vessels are grown from pre-existing vessels. It is controlled by the complicated and coordinated actions of pro-angiogenic and anti-angiogenic factors^1^. Under pathological conditions, angiogenesis is finely regulated by many upregulated angiogenic factors, including ligands and receptors^2^. It is closely associated with various angiogenesis-related diseases, including tumor progression, tumor metastasis, wet age-related macular degeneration, neovascular glaucoma, and diabetic retinopathy^3–6^. We therefore need to elucidate the detailed molecular mechanisms underlying angiogenesis for understanding the progression mechanisms of angiogenesis-related diseases, including cancers.

CLEC14a (C-type lectin domain family 14 member) is a 52-kDa tumor endothelial marker protein that is dominantly expressed on tumor vessels, but not on normal vessels^7^. It is a type I transmembrane protein whose extracellular domain (ECD) contains a C-type lectin-like domain (CLEC14a-CTLD), a sushi-like domain, and an epidermal growth factor-like domain^8^. CLEC14a regulates key angiogenic functions, including filopodia formation, cell-cell adhesion, endothelial cell migration, and tube formation^7–9^. However, we do not yet know the detailed molecular mechanism(s) through which CLEC14a acts in tumor angiogenesis.

Recent studies have suggested that HSP70 is closely associated with tumor progression and metastasis^10–12^. Furthermore, increasing attention is being paid to the drug discovery of HSP70 inhibitors for cancer therapy. More than ten such inhibitors are currently being tested as anti-cancer drugs in pre-clinical and clinical trials. The selective HSP70 inhibitor, MKT-077, exhibits antiproliferative effects on cancer cells but not on normal cells^13, 14^, and shows prominent antitumor activity in mouse xenograft models^15^. More recently, an MKT-077 derivative called YM-1^16^, relevant aptamers (e.g., A8 and A17)^17^, and a mouse monoclonal antibody to the C-terminal epitope of HSP70, called cmHSP70.1^18, 19^, have been developed as potential therapeutic inhibitors of HSP70. Despite the importance of HSP70 as a therapeutic target for cancer therapy, however, the molecular mechanisms underlying its effects in cancer have not yet been intensively studied. Heat shock protein 70-1A (HSP70-1A) is a member of the HSP70 family and is also known as HSPA1A, HSP70-1, HSP72, or HSPA1^20^. Overexpression of HSP70-1A correlates with tumor malignancy and poor survival in several types of cancer^21–24^. Thus, we need to identify and study HSP70–1A-interacting proteins to improve our understanding of the role and regulatory mechanism of HSP70 in cancers.

In this study, we isolated a 70-kDa CLEC14a-CTLD-interacting protein and identified it as HSP70-1A using various proteomic approaches. Our subsequent analyses revealed that HSP70-1A associates specifically with a region comprising amino acids 43 to 69 within CLEC14a-CTLD. Our co-immunoprecipitation experiments verified the interaction between CLEC14a and HSP70-1A on endothelial cells. Finally, using the HSP70-1A-interacting region of CLEC14a-CTLD as a competitor, we validated that the HSP70-1A-CLEC14a interaction promotes angiogenesis by stimulating CLEC14a-CTLD-mediated endothelial cell-cell contacts. Together, our findings suggest that HSP70-1A may be a novel binding partner of CLEC14a-CTLD, and that this interaction could critically regulate HSP70-1A-induced angiogenesis.

## Results

### A 70-kDa protein specifically forms a complex with CLEC14a-CTLD and is identified as HSP70-1A

**Figure 1:**
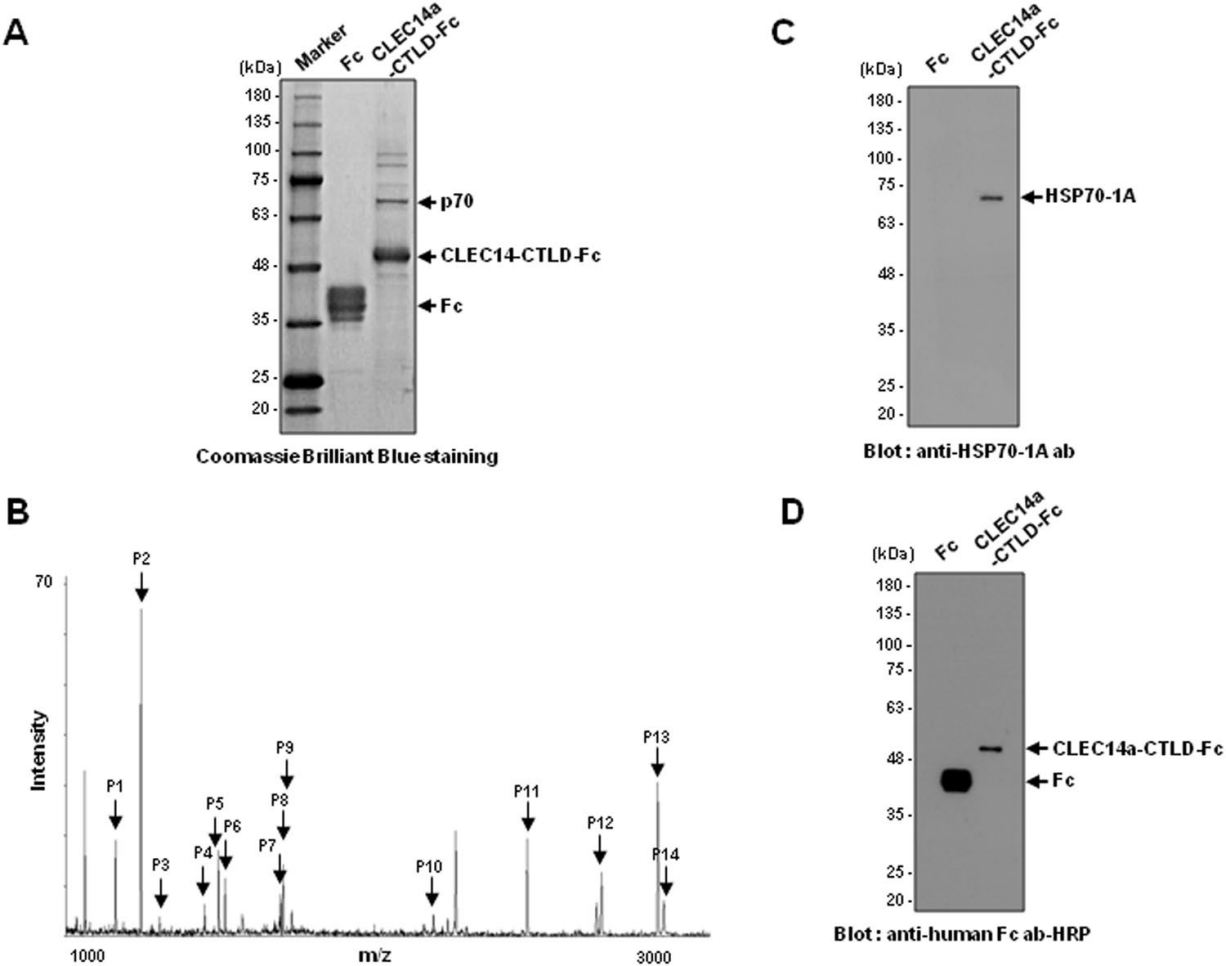
Identification of a 70-kDa CLEC14a-CTLD-binding protein as HSP70-1A. (**A**) HEK293F cells were transfected with vectors encoding CLEC14a-CTLD-Fc or Fc, and after 7 d, the fusion proteins were precipitated from the culture media using protein A Sepharose. The precipitated proteins were separated by SDS-PAGE and visualized with Coomassie brilliant blue staining. The CLEC14a-binding protein with a molecular mass of 70 kDa (p70) is designated by an arrow. Marker: size marker. (**B**) p70 was digested with trypsin, and the resulting peptide mixture was analyzed by matrix-assisted laser desorption ionization/time-of-flight mass spectrometry (MALDI-TOF MS). The arrows (P1–P14) indicate the measured tryptic peaks of p70 whose calculated molecular masses match those of HSP70-1A within 50 ppm. Equal amounts of co-precipitates were separated and analyzed by immunoblotting with antibodies directed against HSP70-1A (**C**) and human Fc (**D**). The lane order is the same as that in (**A**). The results shown are representative of two separate experiments.

We produced CLEC14a-CTLD-Fc and Fc in HEK293F cells and purified the proteins from culture media using affinity column chromatography with protein A Sepharose. We observed that a major protein with a relative molecular mass of 70 (p70) was specifically precipitated with CLEC14a-CTLD-Fc, but not with Fc alone (Fig. 1A). A major band corresponding to p70 in the CLEC14a-CTLD-Fc precipitates was excised from the gel, trypsinized, and subjected to Matrix-assisted Laser Desorption Ionization/Time-of-Flight Mass Spectrometry (MALDI-TOF MS). The masses obtained for the generated peptide fragments, designated P1-P14 (Fig. 1B), were compared with those of proteins in the National Center for Biotechnology Information non-redundant (NCBInr) protein database using the Mascot peptide mass search program. As shown in Supplementary Table S1, the obtained peptides exhibited molecular masses that were almost identical to the calculated masses of theoretically predicted tryptic peptides for HSP70-1A. The peptide mass tolerance was ±0.1 Da, and the analyzed peptides covered 37% of the HSP70-1A sequence.

To further verify the identity of the isolated protein, we confirmed the presence of HSP70-1A in the CLEC14a-CTLD-Fc precipitate by immunoblotting with commercial anti-HSP70-1A antibody that is specific to HSP70-1A (Fig. 1C). The loaded amounts of Fc fusion proteins in the CLEC14a-CTLD-Fc or Fc precipitates were detected by horseradish peroxidase (HRP)-labeled anti-human Fc antibody (Fig. 1D). We found that HSP70-1A was strongly detected in the CLEC14a-CTLD-Fc precipitate but not in the Fc precipitate. To verify the specificity of the antibody, we performed immunoblot analysis using commercially available purified recombinant human HSP70-1A (rhHSP70-1A) and found that the antibody is specific to rhHSP70-1A (Supplementary Fig. S1). Together, these data confirm that the 70-kDa CLEC14a-CTLD-interacting protein is HSP70-1A.

### HSP70-1A specifically interacts with CLEC14a on endothelial cells

**Figure 2:**
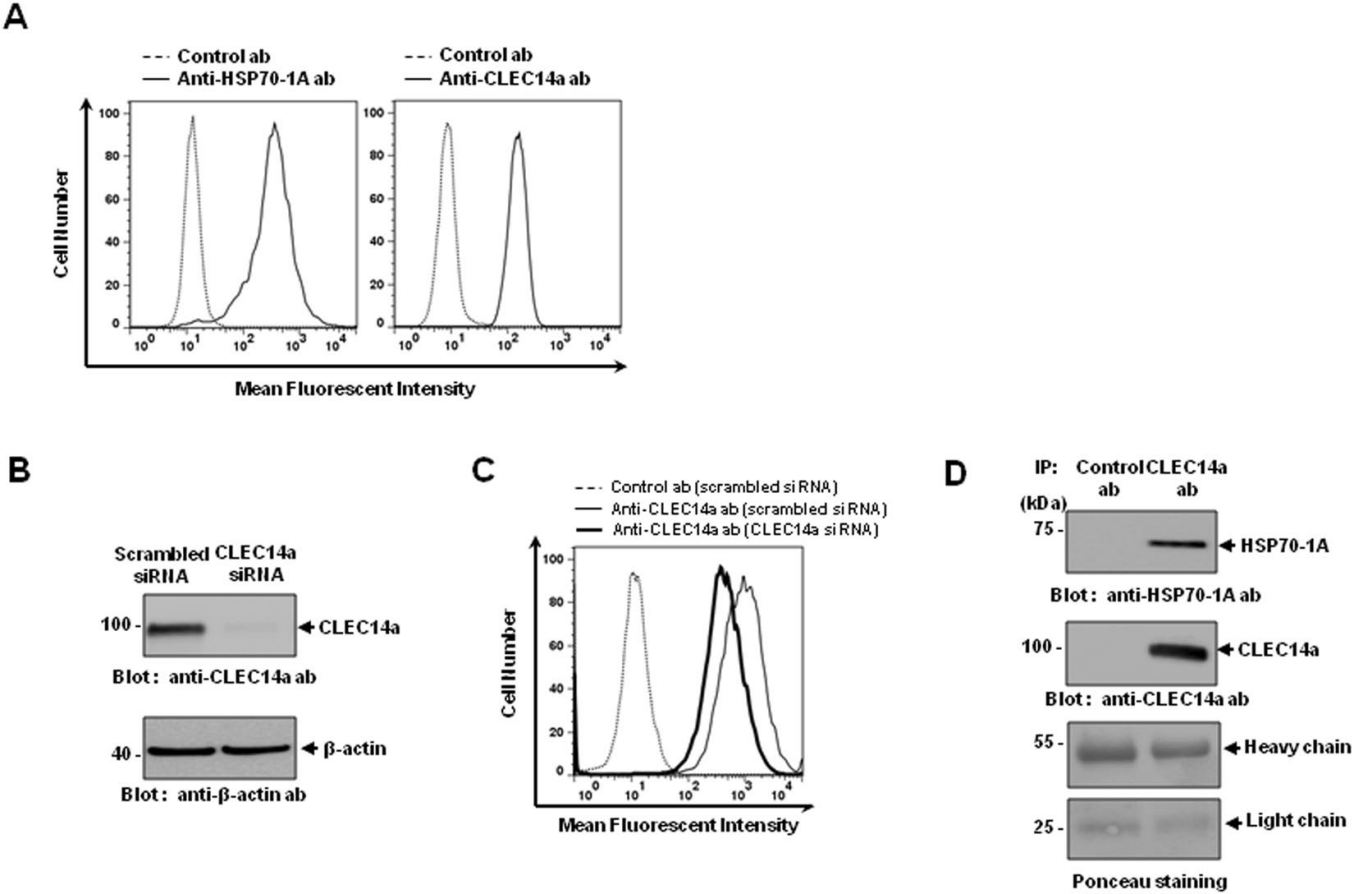
Specific interaction of HSP70-1A with CLEC14a on HUVECs. (**A**) The coexistence of HSP70-1A and CLEC14a on the surfaces of HUVECs was measured by flow cytometry with control antibody (dashed line) or anti-HSP70-1A and anti-CLEC14a antibody (solid line). (**B**) Representative immunoblots show the expression of CLEC14a or β-actin from scrambled siRNA- or CLEC14a siRNA-treated HUVECs. (**C**) CLEC14a on the surface of HUVECs was compared using flow cytometry in presence of control antibody (dashed line) or anti-CLEC14a antibody in scrambled (thin line) siRNA-treated HUVECs or anti-CLEC14a antibody in CLEC14a (thick line) siRNA-treated HUVECs. (**D**) Proteins were co-immunoprecipitated with control antibody or anti-CLEC14a antibody and immunoblotted with anti-HSP70-1A or anti-CLEC14a antibody as designated. The heavy and light chains of the control antibody or anti-CLEC14a antibody were used as a loading control and were stained with Ponceau stain. The results shown are representative of three independent experiments.

To test for the coexistence of HSP70-1A and CLEC14a on the surface of endothelial cells, we first performed flow cytometry with human umbilical vascular endothelial cells (HUVECs) and antibodies against HSP70-1A and CLEC14a. Our results revealed that high amounts of HSP70-1A and CLEC14a are present on the surfaces of HUVECs (Fig. 2A). To investigate the effect of CLEC14a knockdown on HSP70-1A binding to the surface of HUVECs, we first performed siRNA-mediated knockdown of CLEC14a followed by immunoblot analysis and confirmed the specific reduction of CLEC14a expression in CLEC14a siRNA-treated HUVECs (Fig. 2B). Then, using flow cytometry with the commercial anti-HSP70-1A antibody, we found that a considerable amount of HSP70-1A disappeared from the cell surface in CLEC14a siRNA-treated HUVECs compared with scrambled RNA-treated HUVECs (Fig. 2C). To investigate whether HSP70-1A and CLEC14a interact in endothelial cells, we immunoprecipitated HUVEC extracts with anti-CLEC14a or a control antibody. We found that that HSP70-1A co-immunoprecipitated with CLEC14a but was not present in control antibody precipitates (Fig. 2D). These results indicate that HSP70-1A may specifically interact with CLEC14a on the surface of endothelial cells.

### HSP70-1A interacts specifically with CLEC14a-CTLD

**Figure 3:**
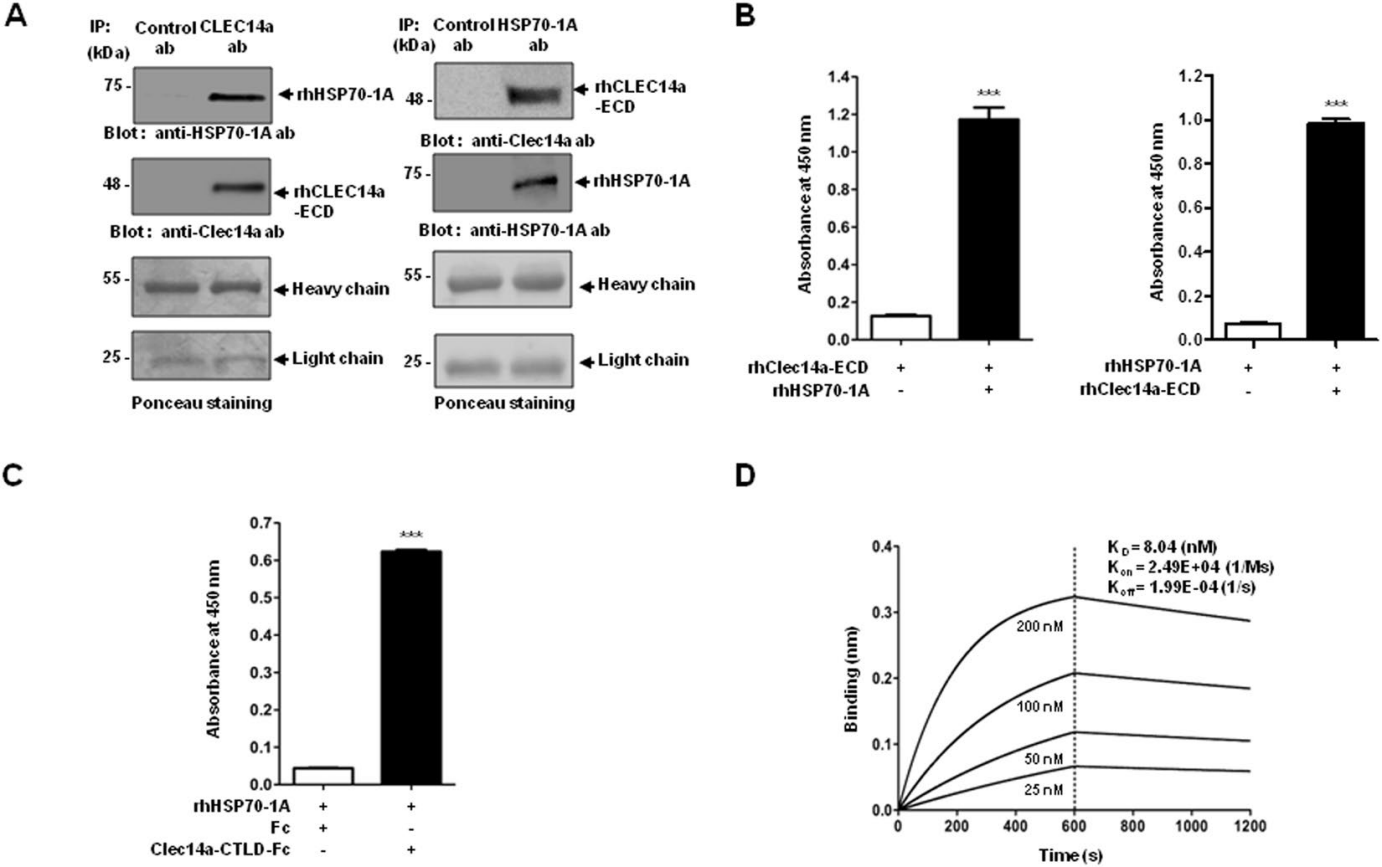
Specific interaction of HSP70-1A with CLEC14a. (**A**) Co-immunoprecipitation with purified rhHSP70-1A or rhCLEC14a-ECD was performed to measure the binding of rhCLEC14a-ECD to rhHSP70-1A (left) or of rhHSP70-1A to rhCLEC14a-ECD (right), respectively. The heavy and light chains of the control antibody, anti-CLEC14a antibody or anti-HSP70-1A antibody were used as a loading control and were stained with Ponceau stain. (**B**) ELISA with purified rhHSP70-1A or rhCLEC14a-ECD was performed to measure the binding of rhHSP70-1A to rhCLEC14a-ECD (left) or of rhCLEC14a-ECD to rhHSP70-1A (right), respectively. (**C**) The bindings of CLEC14a-CTLD-Fc, or Fc to rhHSP70-1A were measured by ELISA. Data are represented as the mean ± SEM of triplicate measurements from two independent experiments; ***P < 0.001. (**D**) The binding affinity of rhHSP70-1A to rhCLEC14a-ECD was measured by a biolayer interferometry assay using an Octet® RED96 system. K_*D*_ = equilibrium dissociation constant; K_on_ = association rate constant; and K_off_ = dissociation rate constant.

To determine whether HSP70-1A associates specifically with CLEC14a, we first performed co-immunoprecipitation in both directions with commercially available purified rhHSP70-1A or recombinant human CLEC14a extracellular domain (rhCLEC14a-ECD). We found that rhHSP70-1A interacts directly with rhCLEC14a-ECD (Fig. 3A). To further investigate the specific interaction between HSP70-1A and CLEC14a, we performed enzyme-linked immunosorbent assay (ELISA) with purified rhHSP70-1A or rhCLEC14a-ECD and measured the interaction using HRP-labeled anti-HSP70-1A antibody. The results indicated that rhHSP70-1A directly bound to rhCLEC14a-ECD (Fig. 3B), suggesting that there is a direct interaction between HSP70-1A and CLEC14a. To examine whether this interaction occurred via the CLEC14a-CTLD, we coated purified rhHSP70-1A onto a 96-well microtiter plate, loaded the wells with CLEC14a-CTLD-Fc or Fc, incubated the plate to allow interaction, and measured the interaction using HRP-labeled anti-human Fc antibody. We found that rhHSP70-1A specifically interacts with CLEC14a-CTLD-Fc, but not Fc alone (Fig. 3C). To more precisely measure the binding affinity of HSP70-1A to CLEC14a-ECD, we performed a label-free kinetic analysis using an Octet biolayer interferometry system. The *K*_d_ for the binding of rhHSP70-1A to rhCLEC14a-ECD was approximately 8 nM (Fig. 3D).

### Amino acids 43-69 of CLEC14a-CTLD are essential for its specific binding to HSP70-1A

**Figure 4:**
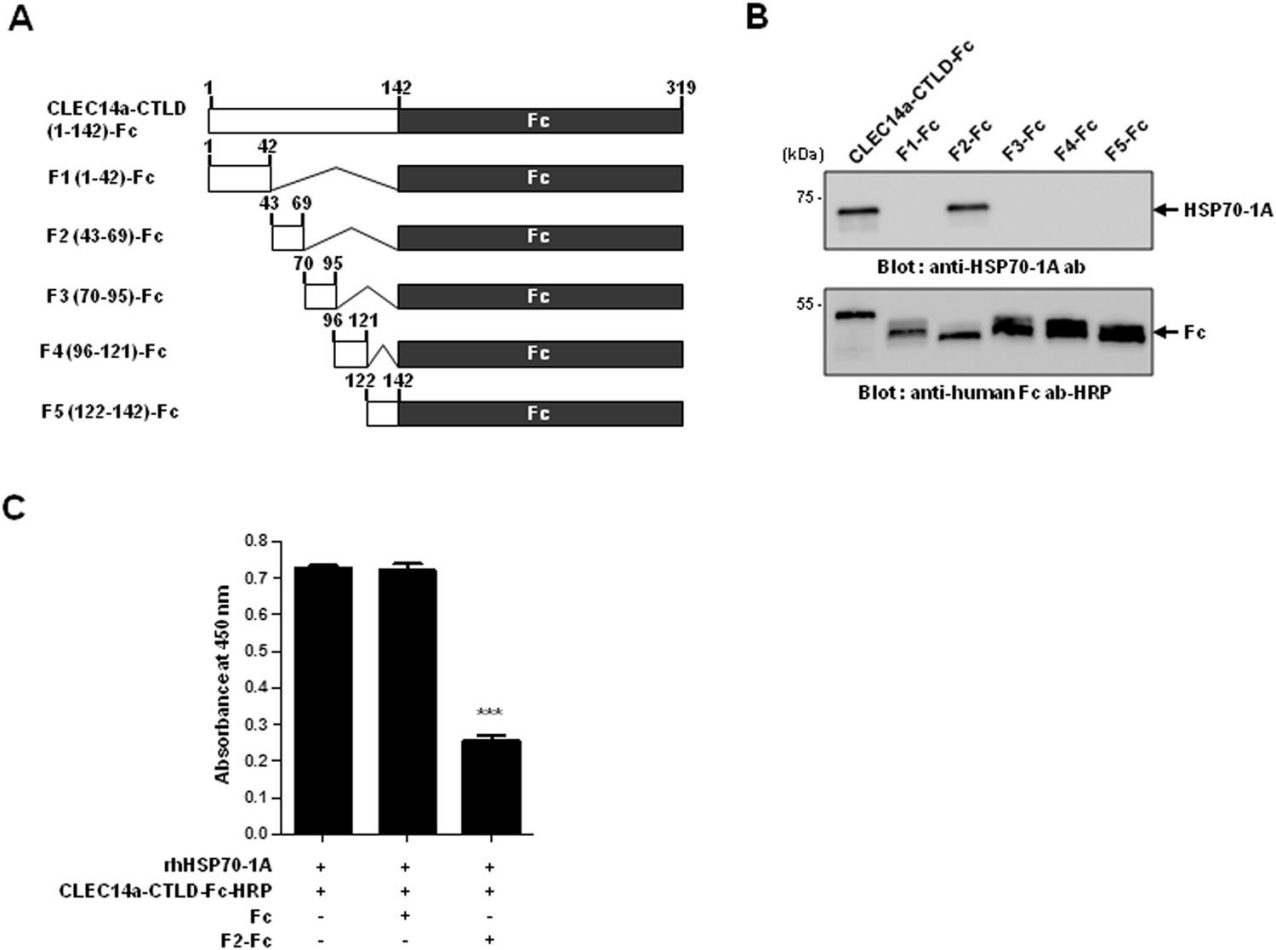
Determination of the HSP70-1A-interacting region of CLEC14a-CTLD. (**A**) Schematic representation of Fc fusion proteins, including CLEC14a-CTLD (amino acids 1–142) and its fragments, F1 (1–42), F2 (42–69), F3 (70–95), F4 (96–121), and F5 (122–142). (**B**) Equal amounts of purified Fc fusion proteins (CLEC14a-CTLD-Fc or F1–F5-Fc) were resolved by PAGE and immunoblotted with antibodies against HSP70-1A (upper panel) and human Fc (lower panel). The results shown are representative of two separate experiments. (**C**) The binding of the CLEC14a-CTLD-Fc-HRP to rhHSP70-1A was measured by ELISA in the absence or presence of F2-Fc or Fc, as indicated. Data are represented as the mean ± SEM of triplicate measurements from two independent experiments; ***P < 0.001.

To identify the CLEC14a-CTLD sequence involved in HSP70-1A binding, we transfected HEK293F cells with constructs encoding various truncated Fc fusion proteins (Fig. 4A) and used affinity column chromatography with protein A Sepharose and subsequent immunoblotting with HRP-labeled anti-HSP70-1A antibody to determine the ability of the generated proteins to bind endogenous HSP70-1A. We found that endogenous HSP70-1A specifically bound to the fragment harboring amino acids 43–69 of CLEC14a-CTLD (F2), but not to the other Fc fusion proteins (Fig. 4B).

To investigate whether the HSP70-1A-interacting region could specifically block the interaction between HSP70-1A and CLEC14a, we pre-coated a 96-well microtiter plate with rhHSP70-1A and performed competitive blocking experiments by applying HRP-labeled CLEC14a-CTLD-Fc (CLEC14a-CTLD-Fc-HRP) in the absence or presence of F2-Fc, or Fc (negative control). Our results revealed that F2-Fc, but not Fc, specifically and significantly reduced the interaction of CLEC14a-CTLD-Fc-HRP with rhHSP70-1A (Fig. 4C). This suggests that F2 could specifically block the interaction between HSP70-1A and CLEC14a-CTLD. Collectively, these results suggest that amino acids 43-96 of CLEC14a, which comprise part of the CTLD, may be important for the specific interaction of CLEC14a with HSP70-1A.

### HSP70-1A specifically increases CLEC14a-CTLD-mediated HUVEC cell-cell contacts

Endothelial cell-cell contact is a prerequisite for the formation of stable blood vessels and a key event in angiogenesis. To elucidate the molecular mechanism through which HSP70-1A acts on endothelial cell-cell contacts in angiogenesis, we established a HUVEC-HUVEC adhesion assay as an indicator of endothelial cell-cell contacts in angiogenesis. We labeled HUVECs with a fluorescent dye and measured the adhesion of dye-labeled HUVECs (i.e., the fluorescent intensity) to an unlabeled HUVEC monolayer in the absence or presence of increasing concentrations of rhHSP70-1A. Our results revealed that HUVEC cell-cell adhesion was dose-dependently increased by rhHSP70-1 (Supplementary Fig. S2A,B), suggesting that HSP70-1A may stimulate endothelial cell-cell contacts in angiogenesis.

**Figure 5:**
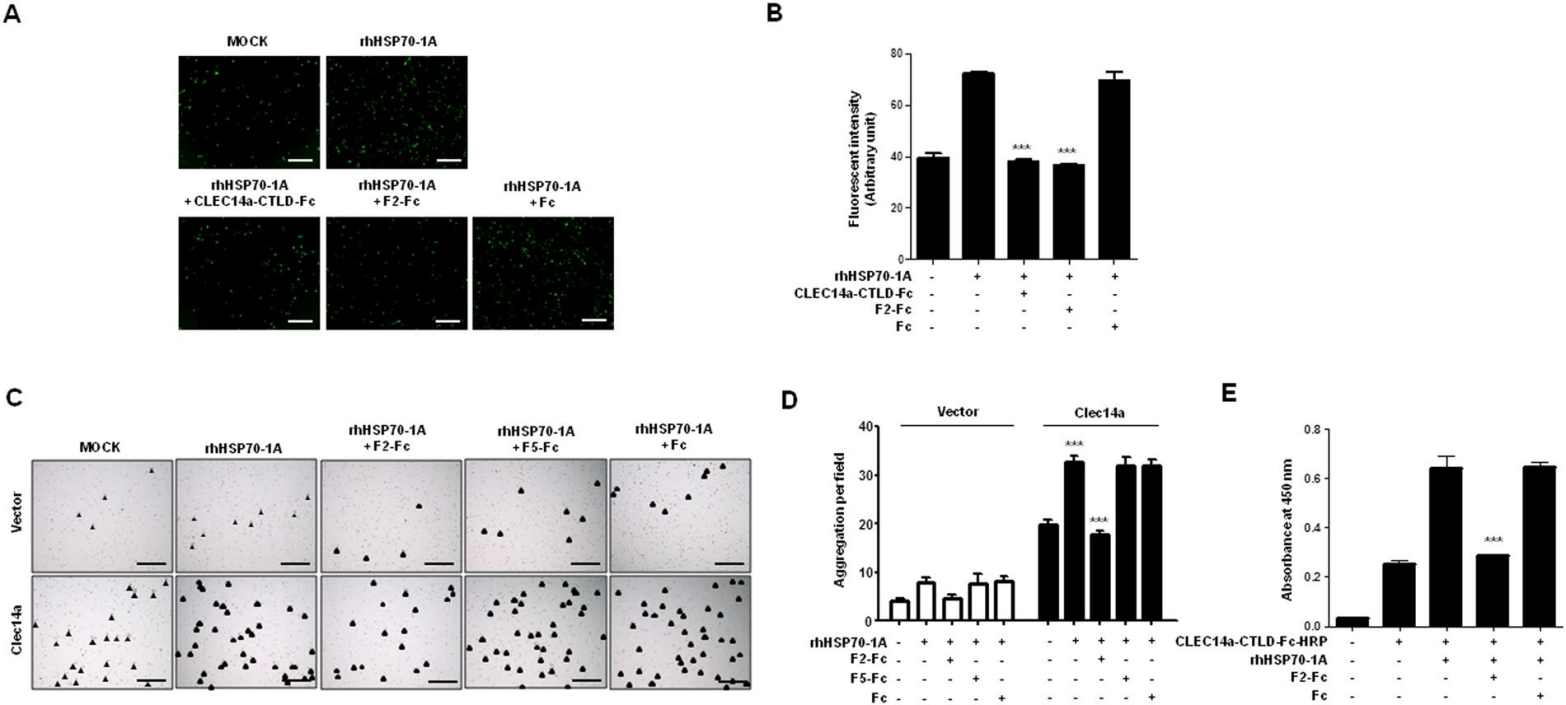
Effect of the HSP70-1A-interacting region of CLEC14a-CTLD on HSP70-1A-induced HUVEC cell adhesion and CLEC14-mediated cell-cell contact. (**A**) Representative images of the binding of calcein-AM-labeled HUVECs to HUVEC monolayers for 2 h in the absence (MOCK) or presence of 50 ng/ml rhHSP70-1A or 50 ng/ml rhHSP70-1A plus 20 μg/ml CLEC14a-CTLD-Fc, F2-Fc, or Fc (scale bar = 400 µm). (**B**) The fluorescence intensities obtained from the calcein-AM labeled HUVECs are expressed as a bar graph. (**C**) HEK293F cells transfected with CLEC14a were incubated in the absence (MOCK) or presence of 50 ng/ml rhHSP70-1A or 50 ng/ml rhHSP70-1A plus F2-Fc, F5-Fc, or Fc for 8 h. Cell aggregates (mass > 4 cells; arrowheads) were counted under a light microscope (scale bar = 500 µm). The number of aggregates per field is shown in. (**D**). (**E**) The binding of CLEC14a-CTLD-Fc-HRP to HUVECs incubated for 2 h in the absence or presence of 50 ng/ml rhHSP70-1A or 50 ng/ml rhHSP70-1A with 20 μg/ml F2-Fc or Fc was measured by cell ELISA. All data are presented as the mean ± SEM of triplicate measurements from two independent experiments; ***P < 0.001.

CLEC14a-CTLD is thought to be a key regulator of endothelial cell-cell contacts in angiogenesis^8, 9^. To elucidate the mechanism through which CLEC14a regulates HSP70-1A-induced angiogenesis, we evaluated the effect of CLEC14a-CTLD-Fc; F2-Fc, an HSP70-1A-interacting region of CLEC14a-CTLD; or Fc alone on HUVEC-HUVEC adhesion in the absence or presence of rhHSP70-1A. Our results indicated that: i) rhHSP70-1A increased HUVEC cell adhesion; and ii) CLEC14a-CTLD-Fc and F2-Fc, but not Fc, specifically and potently reduced rhHSP70-1A-dependent HUVEC cell adhesion (Fig. 5A,B). To further clarify this mechanism, we transfected CLEC14a-negative HEK293F cells with wild-type CLEC14a and then treated the cells with rhHSP70-1A. We then performed a cell aggregation assay, which indicates endothelial cell-cell contact, in the absence or presence of F2-Fc or the negative controls F5-Fc, a non-HSP70-1A-interacting region of CLEC14a-CTLD, or Fc. We found that rhHSP70-1A-induced CLEC14a-mediated cell-cell contact was specifically inhibited by F2-Fc but not by F5-Fc or Fc alone (Fig. 5C,D), further supporting the importance of CLEC14a-HSP70-1A interaction in HSP70-1A-induced angiogenesis. These findings suggest that the HSP70-1A-CLEC14a–CTLD interaction may be critical for endothelial cell-cell contact in HSP70-1A-induced angiogenesis.

To investigate the molecular mechanism through which CLEC14a acts on HSP70-1A-induced angiogenesis, we coated a 96-well microtiter plate with HUVECs, incubated the cells with CLEC14a-CTLD-Fc-HRP, CLEC14a-CTLD-Fc-HRP plus F2-Fc, or CLEC14a-CTLD-Fc-HRP plus Fc in the absence or presence of rhHSP70-1A, and then performed cell ELISA. Similar to CLEC14a-CTLD-Fc, CLEC14a-CTLD-Fc-HRP was found to specifically and significantly bind rhHSP70-1A or rhCLEC14a-ECD (Supplementary Fig. S3A,B). Our results further showed that rhHSP70-1A increased the specific binding of CLEC14a-CTLD-Fc-HRP to the surface of CLEC14a-expressing HUVECs, and that this interaction was specifically inhibited by the addition of F2-Fc, but not by Fc alone (Fig. 5E). Together, these results suggest that the HSP70-1A-CLEC14a-CTLD interaction may stabilize CLEC14-CTLD and subsequently promote the interaction between CLEC14a molecules expressed on adjacent endothelial cells during HSP70-1A-induced angiogenesis.

### Binding of HSP70-1A to CLEC14a-CTLD is critical for HSP70-1A-induced ERK phosphorylation and tube formation in HUVECs

**Figure 6:**
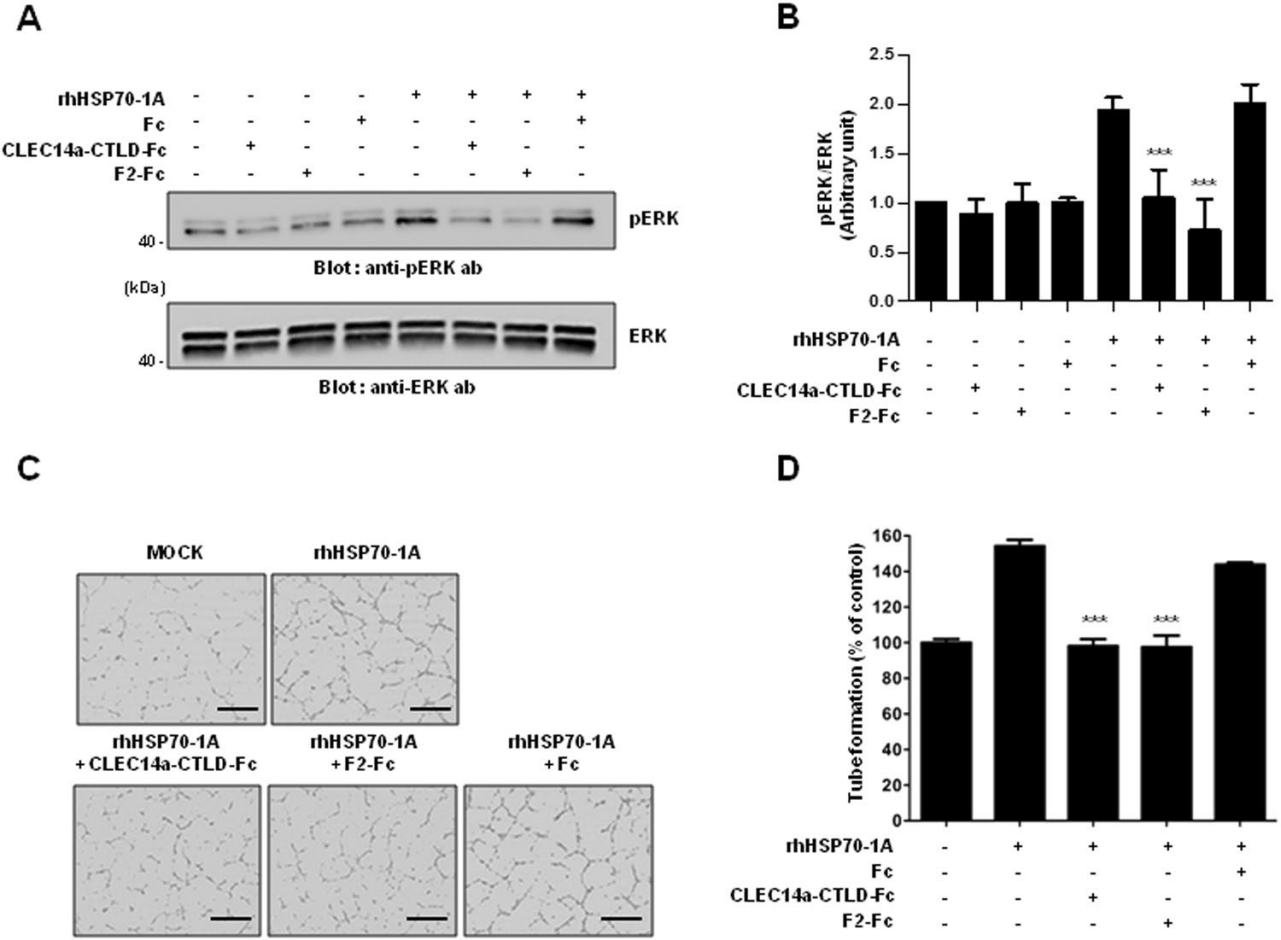
Effect of the HSP70-1A-interacting region of CLEC14a-CTLD on the HSP70-1A-induced ERK phosphorylation and tube formation of HUVECs. (**A**) A representative immunoblot showing the levels of total ERK (lower panel) and phosphorylated ERK (upper panel) in HUVECs incubated for 10 min in the absence (MOCK) or presence of 50 ng/ml rhHSP70-1A or 50 ng/ml rhHSP70-1A plus 20 μg/ml CLEC14a-CTLD-Fc, F2-Fc, or Fc. (**B**) Bar graphs show the mean band densities of pERK normalized with respect to total ERK (pERK/ERK ratio). (**C**) Representative light microscopic images of tubes formed by HUVECs incubated for 6 h in the absence (MOCK) or presence of 50 ng/ml rhHSP70-1A or 50 ng/ml rhHSP70-1A plus 20 μg/ml CLEC14a-CTLD-Fc, F2-Fc, or Fc (scale bar = 500 µm). (**D**) Quantitation of the total number of tube branches, expressed as a percent of control (MOCK) tube formation. All data are presented as the mean ± SEM of quadruplicate measurements from two independent experiments; ***P < 0.001.

ERK is well known to be a central signaling molecule in the regulation of angiogenesis^25^. We previously reported that extracellular HSP70-1A can increase ERK phosphorylation during HSP70-1A-induced angiogenesis^26^. Here, to examine the role of the HSP70-1A-CLEC14a-CTLD interaction in HSP70-1A-induced ERK activation, we performed competitive blocking experiments by treating HUVECs with CLEC14a-CTLD-Fc, F2-Fc, or Fc in the absence or presence of rhHSP70-1A. We then performed immunoblot analysis with anti-phospho ERK to detect the phosphorylation of ERK. Our results showed that CLEC14a-CTLD-Fc and F2-Fc, but not Fc, specifically reduced rhHSP70-1A-dependent ERK phosphorylation (Fig. 6A,B). This indicates that the HSP70-1A-CLEC14a-CTLD interaction is important for HSP70-1A-induced ERK activation.

To investigate the role of the HSP70-1A-CLEC14a-CTLD interaction in HSP70-1A-induced endothelial tube formation, we performed competitive blocking experiments. We treated HUVECs with CLEC14a-CTLD-Fc, F2-Fc, or Fc in the absence or presence of rhHSP70-1A, and then performed tube-formation assays. Our results revealed that CLEC14a-CTLD-Fc and F2-Fc, but not Fc, specifically inhibited rhHSP70-1A-dependent HUVEC tube formation (Fig. 6C,D), indicating that the HSP70-1A-CLEC14a-CTLD interaction is important for HSP70-1A-induced endothelial tube formation. Collectively, our results suggest that the HSP70-1A-CLEC14a-CTLD interaction may be critical for HSP70-1A-induced angiogenesis.

## Discussion

Although increasing evidence supports the functional importance of CLEC14a in tumor angiogenesis, the related regulatory mechanism has not been intensively studied. To this end, we set out to identify a possible CLEC14a-CTLD-binding protein that could be linked to the regulatory mechanism of CLEC14a in angiogenesis. We isolated a 70-kDa CLEC14a-CTLD-interacting protein, identified it as HSP70-1A, and found that the specific binding of HSP70-1A to CLEC14a-CTLD is important for the regulation of HSP70-1A-induced angiogenesis. Our novel findings provide important clues for understanding the mechanism through which CLEC14a regulates HSP70-1A-induced angiogenesis.

Based on our results, we propose for the first time that HSP70-1A may directly interact with a region (amino acids 43–69) of CLEC14a-CTLD on endothelial cells, and that this interaction may be critical for HSP70-1A-induced angiogenesis. Several lines of evidence support this hypothesis. First, we identified a 70-kDa protein that specifically forms a complex with CLEC14a-CTLD. Second, our MALDI-TOF MS and immunoblot analyses identified this CLEC14a-CTLD-interacting protein as HSP70-1A. Third, the results of our co-immunoprecipitation experiments indicated that the two molecules may interact on HUVECs. Fourth, using flow cytometry, we found that HSP70-1A binding to the surface of HUVECs is significantly reduced in CLEC14a siRNA-treated HUVECs compared with scrambled siRNA-treated HUVECs, further supporting the possible interaction of CLEC14a and HSP70-1A on HUVECs. Fifth, our *in vitro* binding analysis with purified proteins demonstrated that HSP70-1A interacts directly with CLEC14a-ECD and more specifically with the region between amino acids 43 and 69 of CLEC14a-CTLD. Sixth, our functional competition assays performed with this HSP70-1A-interacting region of CLEC14a showed that it could specifically and significantly reduce HSP70-1A-induced HUVEC cell-cell adhesion, ERK phosphorylation in HUVECs, and HUVEC tube formation. Seventh, we also found that HSP70-1A stimulates contact between HEK293F cells that overexpress CLEC14a and that this function is specifically blocked by the HSP70-1A-interacting region F2-Fc but not by the HSP70-1A non-interacting region F5-Fc, supporting the specific role of HSP70-1A-CLEC14a interaction in HSP70-1A-induced angiogenesis.

HSP70-1A is a stress-inducible protein that is expressed at low or undetectable levels in most unstressed normal cells and tissues, but is rapidly upregulated under various stressful conditions^27, 28^. HSP70-1A is mainly localized in the cytosol and acts as a molecular chaperone inside cells^29^. During cancer progression and metastasis, the tumor microenvironment causes stressful conditions characterized by oxygen radicals, hypoxia, and nutrient deficiency^30^. The overexpression of HSP70-1A has been reported in a variety of tumors, including liver, prostate, colorectal, breast, lung, ovarian, cervical, oral, bladder, and cervical cancers^31–39^. Although currently it is difficult to directly compare the angiogenic role of secreted versus cytosolic HSP70-1A, accumulating evidence suggests a possible role of extracellular HSP70-1A in tumor angiogenesis. HSP70-1A is secreted from tumor cells^40^, and increased levels of HSP70-1A have been detected in cancer patient blood samples and in the extracellular milieu^41^. In line with our flow cytometric result, there are several reports showing that extracellular HSP70 binds to endothelial cells^42, 43^. Furthermore, HSP70-1A binds to some scavenger receptor (SR) family members, including lectin-like oxidized low-density lipoprotein receptor 1 (LOX1) and SR expressed endothelial cells-1 (SREC-1), which are known to be expressed in endothelial cells^44^. More recently, we employed flow cytometry, cell ELISA, and immunocytochemistry to demonstrate the tight binding of rhHSP70-1A to the surface of HUVECs. Furthermore, we also showed that rhHSP70-1A stimulates HUVEC migration and tube formation *in vitro* in an ERK-dependent manner and microvessel formation *in vivo* to a similar extent as vascular endothelial growth factor (VEGF)^26^, verifying a specific role of extracellular HSP70-1A in angiogenesis. Together, these findings suggest that extracellular HSP70-1A secreted from tumors in response to various stresses may play an important role in tumor angiogenesis; however, we cannot exclude the possibility that other proteins also participate in this process.

Tumor angiogenesis is a hallmark of cancer^45, 46^. Endothelial cell-cell contact is a key event in the formation of stable endothelium for tumor angiogenesis. We recently used anti- CLEC14a-CTLD antibody and CLEC14a-CTLD-deletion mutants to show that CLEC14a-CTLD is a key domain responsible for governing CLEC14a-mediated endothelial cell-cell contacts in angiogenesis^9^. Here, based on many increasing evidences, we hypothesize that the interaction between extracellular HSP70-1A and CLEC14a-CTLD may functionally stabilize CLEC14a expressed on the surface of tumor vessels and promote CLEC14a-mediated endothelial cell-cell contacts in tumor angiogenesis. Traditionally, HSP70-1A acts as a molecular chaperone that facilitates proper protein folding and stabilizes newly synthesized and misfolded proteins^47, 48^. Furthermore, there are several reports showing that HSP70-1A inhibition suppresses angiogenesis. For example, liposomal Quercetin reduces HSP70-1A expression in tumors and results in the efficient suppression of microvessel density and tumor growth^49^. In addition, KNK437 significantly decreases VEGF-induced endothelial migration and tube formation^50^. Previously, we reported that siRNA-mediated knockdown of HSP70-1A reduced HUVEC migration and tube formation^26^. CLEC14a is a tumor endothelial marker protein that is predominantly expressed on tumor vessels, but not on normal vessels^7^. It was previously shown to act as a cell adhesion molecule responsible for mediating endothelial cell-cell contact in angiogenesis^8^, and CLEC14a-knockout mice, a murine syngeneic tumor model, showed that CLEC14a significantly contributes to tumor growth and vascularity^51^. From this study, we demonstrated that HSP70-1A specifically interacts with CLEC14a-CTLD, stimulates CLEC14a-CTLD-mediated endothelial cell-cell contacts, and ultimately participates in HSP70-1A-induced angiogenesis via ERK activation and endothelial tube formation. In addition, we found that our previously generated anti-CLEC14a-CTLD antibody significantly inhibits HSP70-1A-induced HUVEC tube formation (Supplementary Fig. S4). We also found that HSP70-1A-induced HUVEC tube formation was strongly inhibited by siRNA-mediated knockdown of CLEC14a and significantly and additively suppressed by treatment with the HSP70-1A inhibitor VER155008 (Supplementary Fig. S5), suggesting that CLEC14a may be essential for HSP70-1A-induced angiogenesis.

A peptibody is composed of two moieties, a biologically active peptide and an Fc region, and is considered to be an attractive alternative therapeutic format to monoclonal antibodies^52^. Here, we developed the F2-Fc peptibody, which harbored the HSP70-1A-interacting region of CLEC14a-CTLD. This Fc fusion peptide seems to act as an interaction blocker, specifically inhibiting the HSP70-1A-CLEC14a interaction that modulates CLEC14a-mediated endothelial cell-cell contacts to suppress HSP70-1A-induced angiogenesis. We also found that F2-Fc could specifically and significantly inhibit both HSP70-1A-dependent angiogenic functions and endothelial growth medium (EGM)-dependent HUVEC tube formation (Supplementary Fig. S6), leading us to speculate that this Fc fusion peptide may be a useful peptibody platform for suppressing tumor angiogenesis affected by various angiogenic factors. Therefore, this study not only improves our understanding of the mechanism through which CLEC14a-CTLD regulates tumor angiogenesis, it also provides the possibility that the Fc fusion peptide may be useful for efficiently suppressing this process.

**Figure 7:**
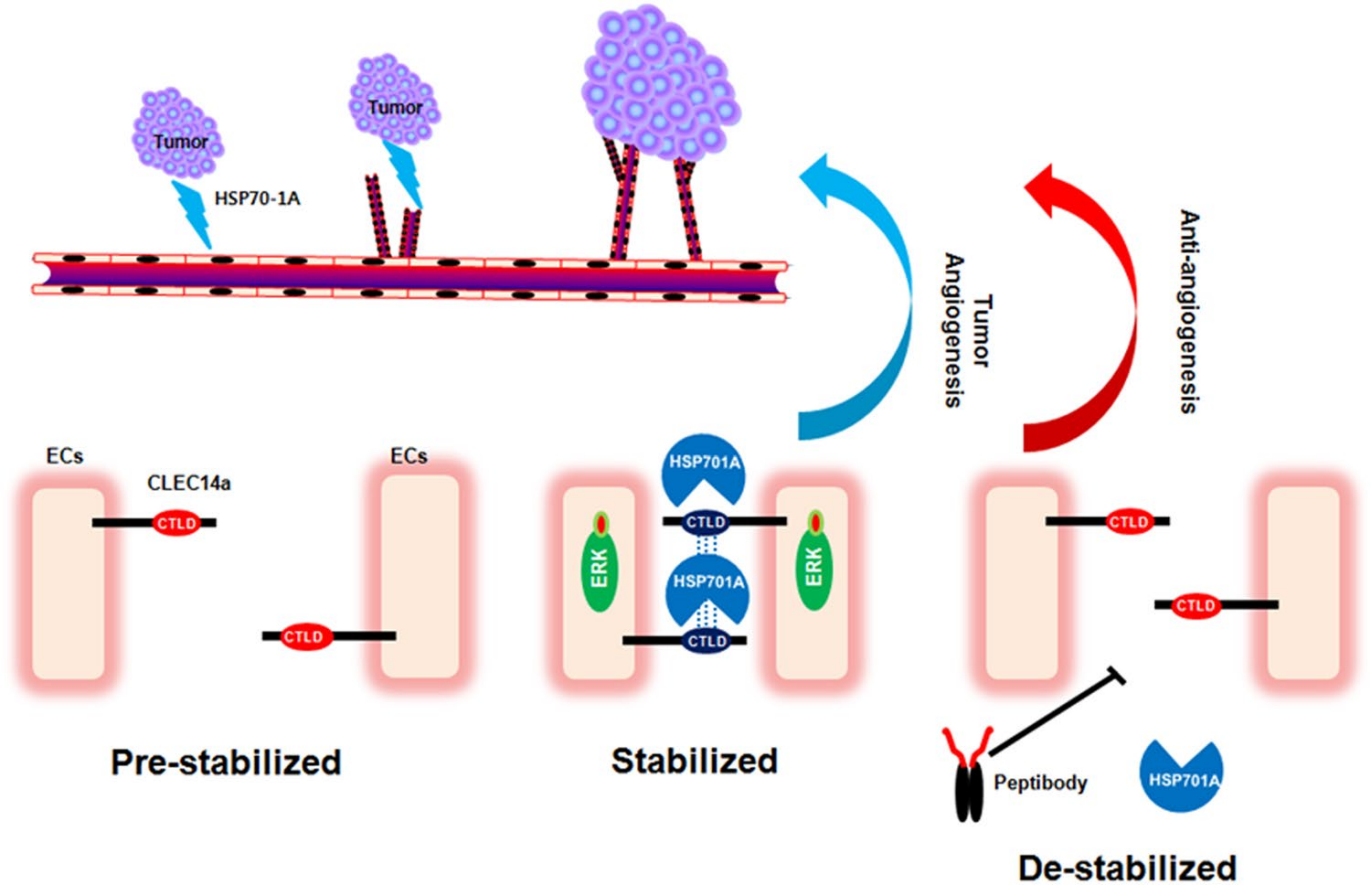
Schematic showing the role of the CLEC14a-CTLD-HSP70-1A interaction in HSP70-1A-induced angiogenesis, and the effect of a peptibody inhibitor of this interaction. When extracellular HSP70-1A secreted from tumor cells specifically binds to the CLEC14a-CTLD of CLEC14a expressed on endothelial cells under pre-stabilized conditions, HSP70-1A stabilizes the conformation of CLEC14a-CTLD and facilitates the CLEC14a-CLEC14a interaction that mediates endothelial cell-cell contact. These interactions stimulate ERK phosphorylation and endothelial tube formation, which are critical for HS70-1A-induced angiogenesis. The application of a peptibody that can specifically block the CLEC14a-CTLD-HSP70-1A interaction will destabilize endothelial cell-cell contacts to inhibit HSP70-1A-induced angiogenesis.

In summary, we herein show for the first time that HSP70-1A is a novel binding partner of CLEC14a, and that this interaction specifically regulates HSP70-1A-induced angiogenesis. On the basis of the currently available evidence, we suggest that depending on spatiotemporal conditions that are linked to tumor angiogenesis within the tumor microenvironment, extracellular HSP70-1A secreted from tumor cells may specifically bind to CLEC14a expressed on tumor vessels via a specific region (amino acids 43–69) of CLEC14a-CTLD to promote CLEC14a-CTLD-mediated endothelial cell-cell contacts and stimulate angiogenic functions. Furthermore, a peptibody we developed seems to inhibit this interaction, resulting in anti-angiogenesis (Fig. 7).

In conclusion, our study suggests a novel regulatory mechanism for CLEC14a-CTLD in HSP70-1A-induced angiogenesis and identifies a new function of CLEC14a-CTLD in facilitating CLEC14a-mediated endothelial cell-cell binding through a specific interaction with HSP70-1A. Furthermore, we also show that the CLEC14a-CTLD-HSP70-1A interaction may be a potential new therapeutic target for suppressing HSP70-1A-induced angiogenesis. In future studies, we plan to plan to develop a human monoclonal antibody to HSP70-1A and further elucidate the detailed molecular mechanism that underlies the effects of the interaction between extracellular HSP70-1A and CLEC14a in tumor-bearing animal models.

## Methods

### Cell culture

Human umbilical vein endothelial cells (HUVECs; Lonza, Basel, Switzerland) were maintained in endothelial growth medium-2 (EGM-2; Lonza) in a humidified incubator (Panasonic Healthcare Company, Tokyo, Japan) at 37 °C with 5% CO_2_. All HUVECs were used at less than passage 5. HEK293F cells (Invitrogen, Carlsbad, CA, USA) were cultured in Freestyle^TM^ expression medium (Invitrogen) in a humidified Multitron incubator shaker (Infors HT, Basel, Switzerland) at 37 °C with 8% CO_2_.

### Isolation of CLEC14a-CTLD-binding proteins

HEK293F cells were transfected with plasmids encoding CLEC14a-CTLD-Fc or Fc as previously described^9^. After 7 days (7 d), the culture medium was collected, and the fusion proteins were collected by affinity column chromatography on protein A Sepharose (GenScript, Piscataway, NJ, USA). The precipitates were resolved by polyacrylamide gel electrophoresis (PAGE) and visualized by Coomassie brilliant blue staining.

### Peptide mass fingerprinting by MALDI-TOF MS

MALDI-TOF MS was performed as described previously^53^. Briefly, the band corresponding to a p70 of the CLEC14a-CTLD-Fc precipitate was excised and digested with trypsin (Roche Molecular Biochemicals, Mannheim, Germany) for 6 h at 37 °C. The masses of the obtained tryptic peptides were determined with a Voyager DE-STR time-of-flight mass spectrometer (Applied Biosystems, Framingham, MA, USA). Delayed ion extraction resulted in peptide masses with better than 50 ppm mass accuracy on average. We then used the amino acid sequences and mass numbers of the tryptic peptides obtained from p70 to search the Swiss-Prot database for a protein match.

### Immunoblot analysis

Immunoblotting was performed as described previously^54^. Briefly, to identify p70 as HSP70-1A, CLEC14a-CTLD-Fc, or Fc precipitates were resolved by SDS-PAGE and transferred to nitrocellulose membranes using a wet transfer system (GE Healthcare Life Sciences, Piscataway, NJ, USA). The membranes were blocked with Tris-buffered saline and Tween [10 mM Tris-HCl, pH 7.5, 150 mM NaCl, and 0.05% (v/v) Tween 20; TBST] containing 5% (w/v) skim milk, and then incubated at 4 °C overnight with mouse anti-HSP70-1A antibody (5A5; 1:1,000; Abcam, Cambridge, MA, USA)^55–57^. For co-immunoprecipitation and determination of the HSP70-1A-interacting site, membranes were incubated with mouse anti-HSP70-1A (1:1,000; Abcam) antibody or sheep anti-CLEC14a antibody (1:1,000; R&D Systems, Minneapolis, MN, USA), respectively. As a loading control, Fc fusion proteins were detected with an HRP-conjugated anti-human Fc antibody (1:5,000; Jackson ImmunoResearch, West Grove, PA, USA). The utilized secondary antibodies included HRP-conjugated goat anti-mouse IgG and donkey anti-sheep IgG (all used at 1:5,000 and obtained from Santa Cruz Biotechnology, Dallas, TX, USA). Following several washings with TBST, the protein bands were visualized using the SuperSignal West Pico Chemiluminescent Substrate (Pierce, Rockford, IL, USA). All immunoblot images are acquired by ImageQuant LAS 4000 mini biomolecular Imager (GE Healthcare Life Sciences).

### Flow cytometry

HUVECs (2 × 10^5^) were fixed in 4% (v/v) paraformaldehyde (PFA) for 20 min at room temperature. The cells were blocked with PBS containing 1% (w/v) bovine serum albumin (BSA) for 1 h at room temperature, incubated with mouse anti-HSP70-1A (1:100; Abcam) or sheep anti-CLEC14a antibody (1:10; R&D Systems) for 1 h at 37 °C, and then incubated with Alexa Fluor 488-conjugated anti-mouse (1:1,000; Invitrogen) or Alexa Fluor 546-conjugated anti-sheep antibody (1:1,000; Invitrogen), respectively, for 1 h at 37 °C. Samples were analyzed by flow cytometry using a FACSCalibur system (BD Biosciences, San Jose, CA, USA) with the aid of the FlowJo software (TreeStar, Ashland, OR, USA).

### Co-immunoprecipitation

HUVECs were lysed in ice-cold lysis buffer containing 1% (v/v) TX-100 and a protease inhibitor cocktail (GenDEPOT, Barker, TX, USA) in PBS. The lysates were briefly sonicated, incubated for 1 h with constant agitation, and then centrifuged at 100,000 × g for 1 h. Each cell extract (1 mg of proteins) was incubated overnight at 4 °C with 1 μg of control antibody or sheep anti-CLEC14a antibody (R&D Systems) immobilized on protein G resin (EMD Millipore, Billerica, MA, USA). To detect the interaction between HSP70-1A and CLEC14a, 0.3 μg of purified rhHSP70-1A (Sino Biological Inc., Beijing, China) or rhCLEC-14a-ECD (Abcam) were incubated overnight at 4 °C with 1 μg of anti-HSP70-1A antibody (Abcam) or anti-CLEC14a antibody (R&D Systems) immobilized on protein G resin. Then, binding partners were individually incubated with an equal molar concentration of purified rhCLEC-14a-ECD or rhHSP70-1A for 3 h at room temperature. After three times washings with PBS containing 0.1% (v/v) TX-100, the precipitates were loaded onto a polyacrylamide gel for immunoblot analysis.

### ELISA

ELISA was performed as described previously^9^. Briefly, to determine the binding of HSP70-1A to CLEC14a-ECD, 96-well plates were coated with 0.1 µg/well of rhCLEC14a-ECD (Abcam) or rhHSP70-1A (Sino Biological Inc), incubated overnight at 37 °C, washed twice with PBS containing 0.05% (v/v) Tween 20 (PBST), incubated with blocking buffer containing 3% (w/v) BSA in PBST for 1 h at 37 °C, and then incubated with 1 µg of rhHSP70-1A or rhCLEC14a-ECD in blocking buffer for 2 h at 37 °C. The plates were washed three times with PBST and then incubated with HRP-conjugated anti-HSP70-1A antibody (1:1,000; Abcam) or anti-CLEC14a antibody (R&D Systems) in blocking buffer for 1 h at 37 °C. For the detection of the binding of the anti-CLEC14a antibody, HRP-conjugated donkey anti-sheep IgG (Santa Cruz Biotechnology) was used as the secondary antibody. To examine the binding of HSP70-1A to CLEC14a-CTLD, 96-well plates were first coated with 0.1 µg/well of rhHSP70-1A and then incubated with 1 µg of CLEC14a-CTLD-Fc or Fc alone in blocking buffer for 2 h at 37 °C. Following three times washings with PBST, the plates were incubated with HRP-conjugated anti-human Fc antibody (1:5,000; Jackson ImmunoResearch, West Grove, PA, USA) in blocking buffer for 1 h at 37 °C. To perform a competition assay with F2-Fc and Fc, we coated a 96-well plate with 1 µg/well of rhHSP70-1A and incubated 0.5 µg/well of CLEC14a-CTLD-Fc-HRP in the absence or presence of 4 µg F2-Fc or Fc for 2 h at 37 °C. Following three times washings with PBST, the plates were incubated with HRP-conjugated anti-human Fc antibody (1:5,000; Jackson ImmunoResearch) in blocking buffer for 1 h at 37 °C. For colorimetric measurement, 3,3′,5,5′-tetramethylbenzidine substrate solution (TMB; BD Biosciences) was added to each well and the reactions stopped by adding an equal volume of 1 N H_2_SO_4_ to each well. Optical density at 450 nm was measured using a microplate reader (VICTOR × 4; PerkinElmer).

### Cell ELISA

HUVECs (10^4^ per well) were loaded to a 96-well microtiter plate, and then incubated for 2 h at 37 °C with 0.3 μg of CLEC14a-CTLD-Fc-HRP in the absence or presence of 2 μg rhHSP70-1A, rhHSP70-1A plus 2 μg F2-Fc, or rhHSP70-1A plus Fc. The plates were washed twice with PBST, and TMB was added to each well. Optical density was measured at 450 nm using a microplate reader (VICTOR × 4; PerkinElmer).

### Real-time measurement of HSP70-1A–CLEC14a-CTLD interactions

A biolayer interferometry (BLI) assay was performed using an Octet^®^ RED96 system (ForteBio/Pall Life Sciences, Menlo Park, CA, USA) as described previously^58^. Briefly, following the immobilization of rhCLEC14a-ECD onto amine-reactive biosensors (ForteBio), rhHSP70-1A was serially diluted 2-fold (200–25 nM) in 1 × kinetics buffer. The association and dissociation (*K*_d_) constants were determined by 1:1 binding-model fitting using the ForteBio Octet Data Analysis software version 7.1 (ForteBio).

### Preparation of Fc fusion proteins and mapping of the HSP70-1A-interacting site

The DNA sequences encoding CLEC14a-CTLD and fragments F1–F5 were amplified using polymerase chain reaction (PCR). The PCR products were subcloned to the two asymmetric *Sfi* I sites of a modified pCEP4 mammalian expression vector (Invitrogen) that contains the hinge and CH2-CH3 domains of human IgG1 at the 3′ end of the cloning site. Following producing each fragment in HEK293F cells, the fusion proteins were purified from the culture media using affinity chromatography with protein A Sepharose beads (GenScript). To determine the region through which HSP70-1A interacts with CLEC14a-CTLD, 0.1 µg of Fc fusion protein (CLEC14a-CTLD or F1–F5) were loaded onto a polyacrylamide gel for immunoblotting.

### Endothelial cell-cell adhesion assay

Endothelial cell-cell contacts were performed as described previously with minor modifications^54^. Briefly, HUVECs (10^4^ per well) labeled with 5 µM calcein-AM (Molecular Probes, Life Technologies, Grand Island, NJ, USA) in endothelial basal medium (EBM) were added to a HUVEC monolayer and incubated for 2 h at 37 °C in the presence of EBM alone (MOCK), EBM containing 50 ng/ml rhHSP70-1A, or EBM containing 50 ng/ml rhHSP70-1A plus 20 µg/ml CLEC14a-CTLD-Fc, F2-Fc, or Fc. The adherent cells were measured using an IncuCyte FLR live content imaging system (Essen Bioscience Inc.). Quantification was performed by measuring fluorescence with excitation (470 nm) and emission (515 nm) filters.

### Cell aggregation assay

Cell aggregation assays were performed as described previously^9^. Briefly, 1.5 × 10^7^ HEK293F cells in suspension were transfected with plasmids for expression of CLEC14a, cultured in Freestyle 293 expression medium overnight, and then seeded in 6-well plates (5 × 10^5^ cells/well). Cells were maintained in the presence or absence of 50 ng/ml rhHSP70-1A or 50 ng/ml rhHSP70-1A plus 20 µg/ml F2-Fc, F5-Fc, or Fc for 8 h. Cell aggregates (mass >4 cells) were counted in at least 3 fields.

### Measurement of ERK phosphorylation

HUVECs (3 × 10^5^) plated to a 6-well microtiter plate were incubated for 30 min at 37 °C in EBM (MOCK), EBM containing 20 ng/ml rhHSP70-1A, or EBM containing 20 ng/ml rhHSP70-1A plus 20 μg/ml CLEC14a-CTLD-Fc, F2-Fc, or Fc. The samples were then washed twice with ice-cold PBS and lysed with ice-cold lysis buffer. Total cell lysates (10 μg) were then individually loaded onto a polyacrylamide gel for immunoblotting.

### Tube-formation assay

Tube-formation assays were performed as described previously^9^. Briefly, 150 µl Matrigel (Corning, Tewksbury, MA, USA) was added to each well of a 48-well plate and allowed to polymerize for 30 min at 37 °C. HUVECs (10^5^ per well) were seeded to the Matrigel-coated plate and incubated for 6 h at 37 °C in EBM alone (MOCK), EBM containing 20 ng/ml rhHSP70-1A, or EBM containing 20 ng/ml rhHSP70-1A plus 20 µg/ml CLEC14a-CTLD-Fc, F2-Fc, or Fc. Images were obtained using an IncuCyte FLR live content imaging system (Essen Bioscience Inc., Ann Arbor, MI, USA), and tube formation was quantified by counting the total tube branches.

### Statistical analysis

All data were analyzed with GraphPad Prism 5.0 (GraphPad Software, La Jolla, CA, USA). We used a two-tailed Student’s *t*-test for comparisons between two groups, and a one-way analysis of variance with Bonferroni’s correction for multiple comparisons. All data are presented as the mean ± the standard error of the mean (SEM). *P* < 0.05 was considered statistically significant.

## Electronic supplementary material

Supplementary information
