# Supplementary material for: CLEC14a-HSP70-1A interaction regulates HSP70-1A-induced angiogenesis
Source: Sci Rep. 2017 Sep 6;7:10666. doi: 10.1038/s41598-017-11118-y (PMC5587741; doi:10.1038/s41598-017-11118-y)
Supplement: Supplementary file 1 — Supplementary information [file 41598_2017_11118_MOESM1_ESM.pdf]

CLEC14a-HSP70-1A interaction regulates HSP70-1A-induced angiogenesis

Jihye Jang, Mi Ra Kim, Taek-Keun Kim, Woo Ran Lee, Jong Heon Kim, Kyun Heo, and Sukmook Lee*

Supplementary Figure legends

Supplementary Figure S1: Reactivity of commercial anti-HSP70-1A antibody to rhHSP70-1A. Following the separation of 0.1 μg rhHSP70-1A by SDS-PAGE, the reactivity of the commercial anti-HSP70-1A antibody was measured via immunoblotting.

Supplementary Figure S2: Effect of rhHSP70-1A on HUVEC cell adhesion. (**A**) Representative fluorescent microscopic images of calcein AM-labeled HUVECs bound to HUVEC monolayers in the absence or presence of the indicated increasing concentrations of rhHSP70-1A (scale bar=400 μm). (**B**) Fluorescence intensity of the calcein-AM labeled HUVECs is expressed as a bar graph. The data represent the mean ± SEM of triplicate measurements from two independent experiments; **P < 0.01, ****P* < 0.001.

Supplementary Figure S3: Measurement of the interaction between CLEC14a-CTLD-Fc-HRP and rhHSP70-1A or rhCLEC14a-ECD. A 96-well microtiter plate was coated with 0.1 μg of rhHSP70-1A (**A**) or rhCLEC14a-ECD (**B**) per well, loaded with 1 μg/well of CLEC14a-CTLD-Fc-HRP, and incubated for 2 h. Binding to the coated protein was assessed by ELISA. Data are presented as the mean ± SEM of triplicate measurements from two independent experiments; ****P* < 0.001.

Supplementary Figure S4: Effect of the anti-CLEC14a-CTLD antibody on HSP70-1A-induced HUVEC tube formation and HSP70-1A-CLEC14a interaction. (**A**) Representative light microscopic images of tubes formed by HUVECs incubated for 8 h with 50 ng/ml rhHSP70-1A or 50 ng/ml rhHSP70-1A plus 20 μg/ml control IgG or anti-CLEC14a-

CTLD antibody (scale bar=500 μm). HUVECS incubated in the absence of rhHSP70-1A were used as controls (MOCK). (**B**) Quantitation of the total number of tube branches, expressed as a percent of control (MOCK) tube formation. (**C**) Wells of a 96-well plate were coated with 0.1 μg of rhCLEC14a-ECD followed by the addition of 1 μg of rhHSP70-1A. ELISA was performed in the absence (MOCK) or presence of 20 μg/ml control IgG or anti-CLEC14a-CTLD antibody. The data represent the mean ± SD of triplicate experiments. ***P < 0.001.

Supplementary Figure S5: Effect of VER155008 or siRNA-mediated knockdown of CLEC14a on HSP70-1A-induced HUVEC tube formation. (**A**) Representative light microscopic images of tubes formed following treatment of HUVECs treated with scrambled- or CLEC14a siRNA. HUVECs were incubated for 8 h in the absence (MOCK) or presence of 20 nM VER155008, 50 ng/ml rhHSP70-1A or 50 ng/ml rhHSP70-1A plus 20 nM VER155008 (scale bar=500 μm). (**B**) Quantitation of the total number of tube branches, expressed as a percent of control (MOCK) tube formation. The data represent the mean ± SD of triplicate experiments. *$P < 0.05$, **P < 0.01, ***$P < 0.001$.

Supplementary Figure S6: Effect of the HSP70-1A-interacting region of CLEC14a-CTLD on EGM-induced HUVEC tube formation. (**A**) Representative light microscopic images of tubes formed by HUVECs incubated for 12 h in the absence (MOCK) or presence of EGM or EGM plus 20 μg/ml CLEC14a-CTLD-Fc, F2-Fc, F5-Fc, or Fc (scale bar=500 μm). (**B**) Quantitation of the total number of tube branches, expressed as a percent of control (MOCK) tube formation. The data represent the mean ± SEM of quadruplicate measurements from two independent experiments; ***$P < 0.001$.

Supplementary Figure S7: Full-length images of the cropped blots throughout this study. (**A**) Immunoblot images depicting the expression of CLEC14a (left) or β-actin (right) from scrambled siRNA- or CLEC14a siRNA-treated HUVECs shown in Fig. 2B. (**B**) Immunoblot images depicting the co-immunoprecipitated HSP70-1A (left) or CLEC14a (right) shown in in Fig. 2D. (**C**) Ponceau stained images depicting the amount of heavy and light chains of control antibody or anti-CLEC14a antibody shown in Fig. 2D. (**D**) Immunoblot images depicting the co-immunoprecipitated rhCLEC14a-ECD (left) or rhHSP70-1A (right) shown in Fig. 3A (left). (**E**). Ponceau stained images depicting the amount of heavy and light chains of control antibody or anti-CLEC14a antibody shown in Fig. 3A (left). (**F**) Immunoblot images depicting the co-immunoprecipitated rhCLEC14a-ECD (left) or rhHSP70-1A (right) shown in Fig. 3A (right). (**G**) Ponceau stained images depicting the amount of heavy and light chains of control antibody or anti-HSP70-1A antibody shown in Fig. 3A (right). (**H**) Immunoblot images depicting the amount of HSP70-1A bound to CLEC14a-CTLD-Fc or Fc fusion fragments (left) shown in the upper panel of Fig. 4B. Immunoblot images depicting the loaded amounts of CLEC14a-CTLD-Fc or Fc fusion fragments (right) shown in the lower panel of Fig. 4B. (**I**) Immunoblot images depicting the levels of phosphorylated ERK (left) or total ERK (right) shown in Fig. 6A.

Supplementary Table S1 Peptide sequences and masses from p70 by MALDI TOF

Peptide	Sequence[a]	M + H$^+$	
		Observed	Calculated[b]
		Da	
P1	LLQDFFNGR (349-357)	1109.55	1108.56
P2	DAGVIAGLNVLR (160-171)	1197.66	1196.68
P3	LVNHFVEEFK (237-246)	1261.65	1260.65
P4	LVNHFVEEFKR (237-247)	1417.72	1416.75
P5	AQIHDLVLVGGSTR (329-342)	1464.77	1464.80
P6	TTPSYVAFTDTER (37-49)	1487.66	1486.69
P7	ATAGDTHLGGEDFDNR (221-236)	1675.69	1674.72
P8	HWPFQVINDGDKPK (89-102)	1680.83	1679.84
P9	IINEPTAAAIAYGLDR (172-187)	1687.87	1686.89
P10	AAAIGIDLGTTYSCVGVFQHGK (4-25)	2208.06	2207.10
P11	GVPQIEVTFDIDANGILNVTATDK (470-493)	2530.25	2529.29
P12	QTQIFTTYSDNQPGVLIQVYEGER (424-447)	2786.34	2785.35
P13	TLSSSTQASLEIDSLFEGIDFYTSITR (273-299)	2981.43	2980.45
P14	EIAEAYLGYPVTNAVITVPAYFNDSQR (129-155)	3001.48	3000.48

[a] The matched peptides cover 37% (239 of 641 amino acids) of the proteins.

[b] Monoisotopic mass.

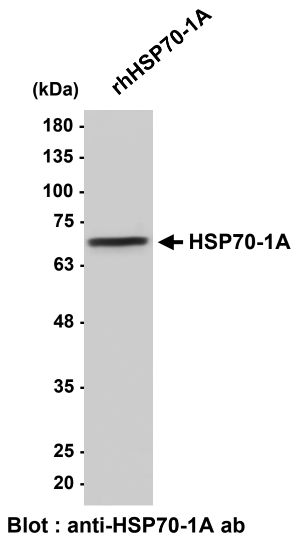

rhHSP70-1A
(kDa)
180
135
100
75
63
48
35
25
20
HSP70-1A
Blot : anti-HSP70-1A ab

Fig. S1

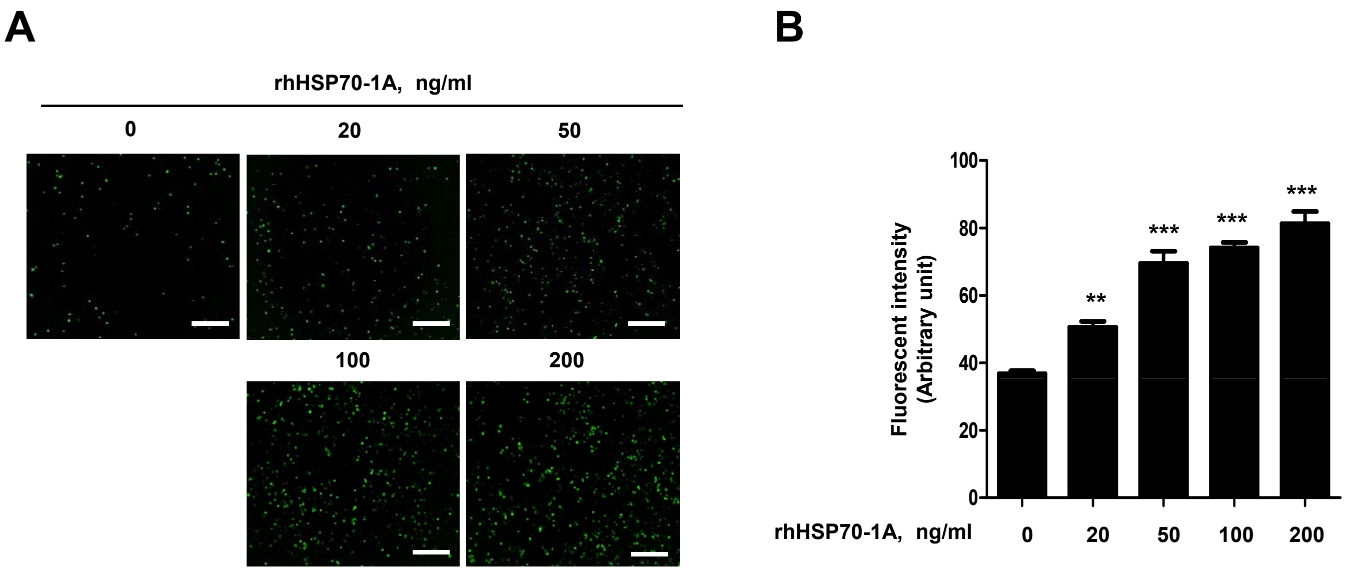
A
rhHSP70-1A, ng/ml
0
20
50
100
200
B
Fluorescent intensity
(Arbitrary unit)
100
80
60
40
20
0
**
***
***
***
rhHSP70-1A, ng/ml
0
20
50
100
200

Fig. S2

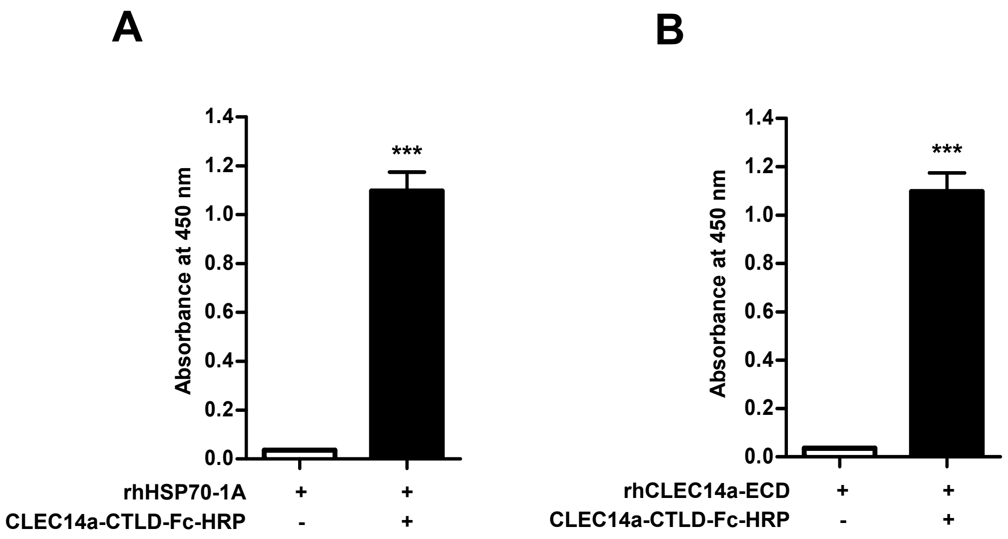
A
B
Absorbance at 450 nm
1.4
1.2
1.0
0.8
0.6
0.4
0.2
0.0
***
rhHSP70-1A + +
CLEC14a-CTLD-Fc-HRP - +
Absorbance at 450 nm
1.4
1.2
1.0
0.8
0.6
0.4
0.2
0.0
***
rhCLEC14a-ECD + +
CLEC14a-CTLD-Fc-HRP - +

Fig. S3

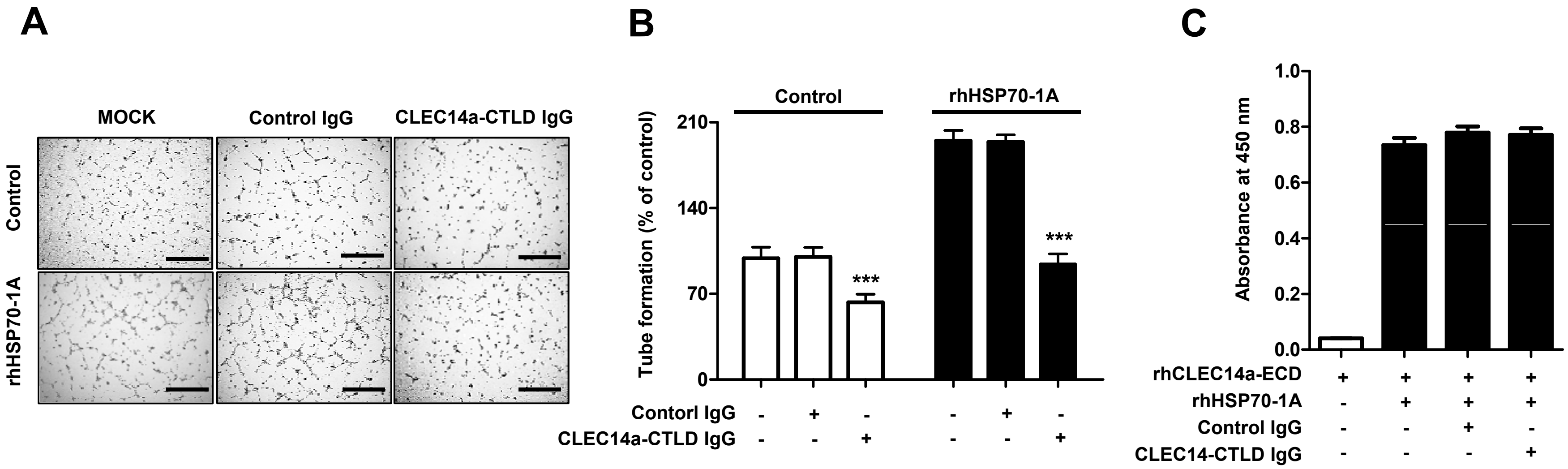
A
MOCK
Control IgG
CLEC14a-CTLD IgG
Control
rhHSP70-1A
B
Control
rhHSP70-1A
Tube formation (% of control)
210
140
70
0
***
***
Contorl IgG - + - - + -
CLEC14a-CTLD IgG - - + - - +
C
Absorbance at 450 nm
1.0
0.8
0.6
0.4
0.2
0.0
rhCLEC14a-ECD + + + +
rhHSP70-1A - + + +
Control IgG - - + -
CLEC14-CTLD IgG - - - +

Fig. S4

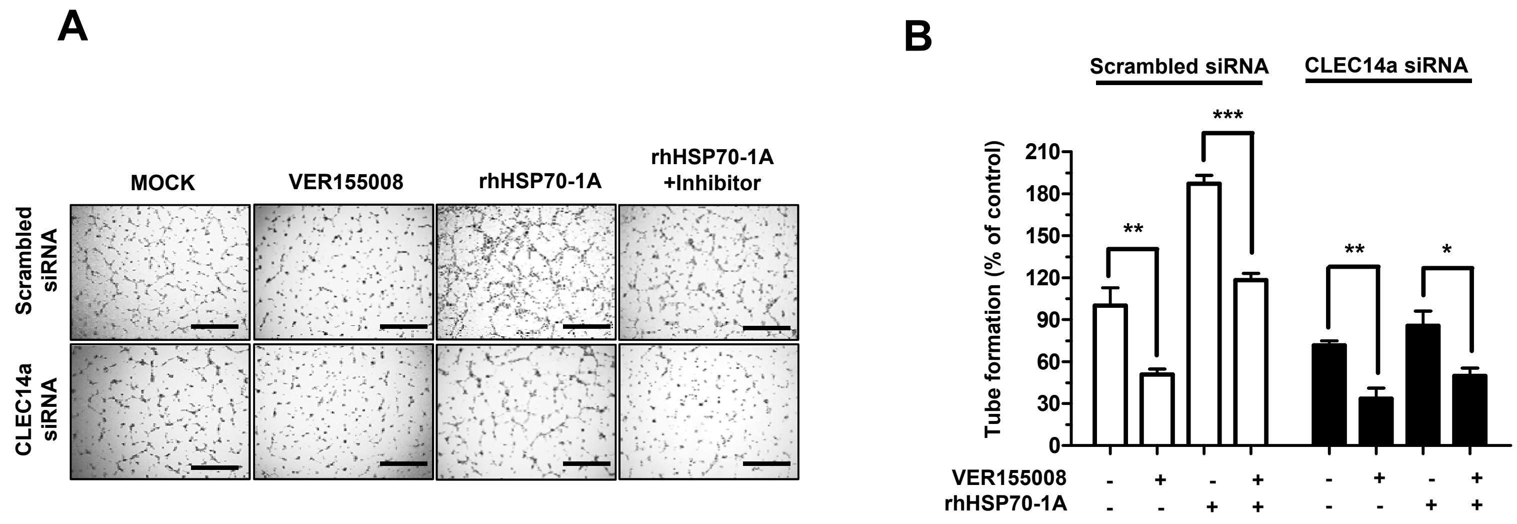
A
MOCK
VER155008
rhHSP70-1A
rhHSP70-1A +Inhibitor
Scrambled siRNA
CLEC14a siRNA
B
Scrambled siRNA
CLEC14a siRNA
Tube formation (% of control)
210
180
150
120
90
60
30
0
**
***
**
*
VER155008
rhHSP70-1A

Fig. S5

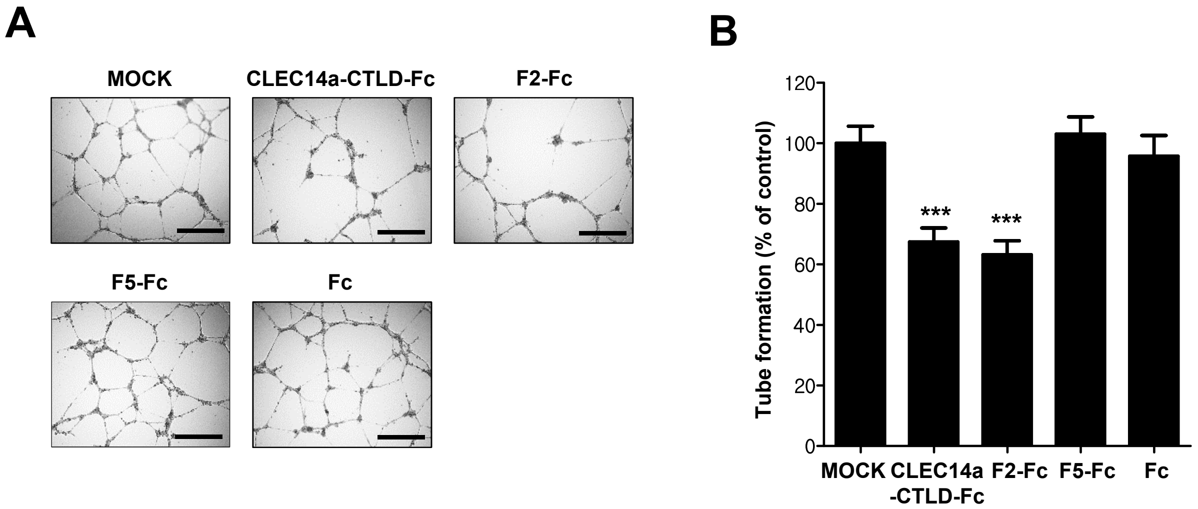
A
MOCK
CLEC14a-CTLD-Fc
F2-Fc
F5-Fc
Fc
B
Tube formation (% of control)
0
20
40
60
80
100
120
***
***
MOCK
CLEC14a
-CTLD-Fc
F2-Fc
F5-Fc
Fc

Fig. S6

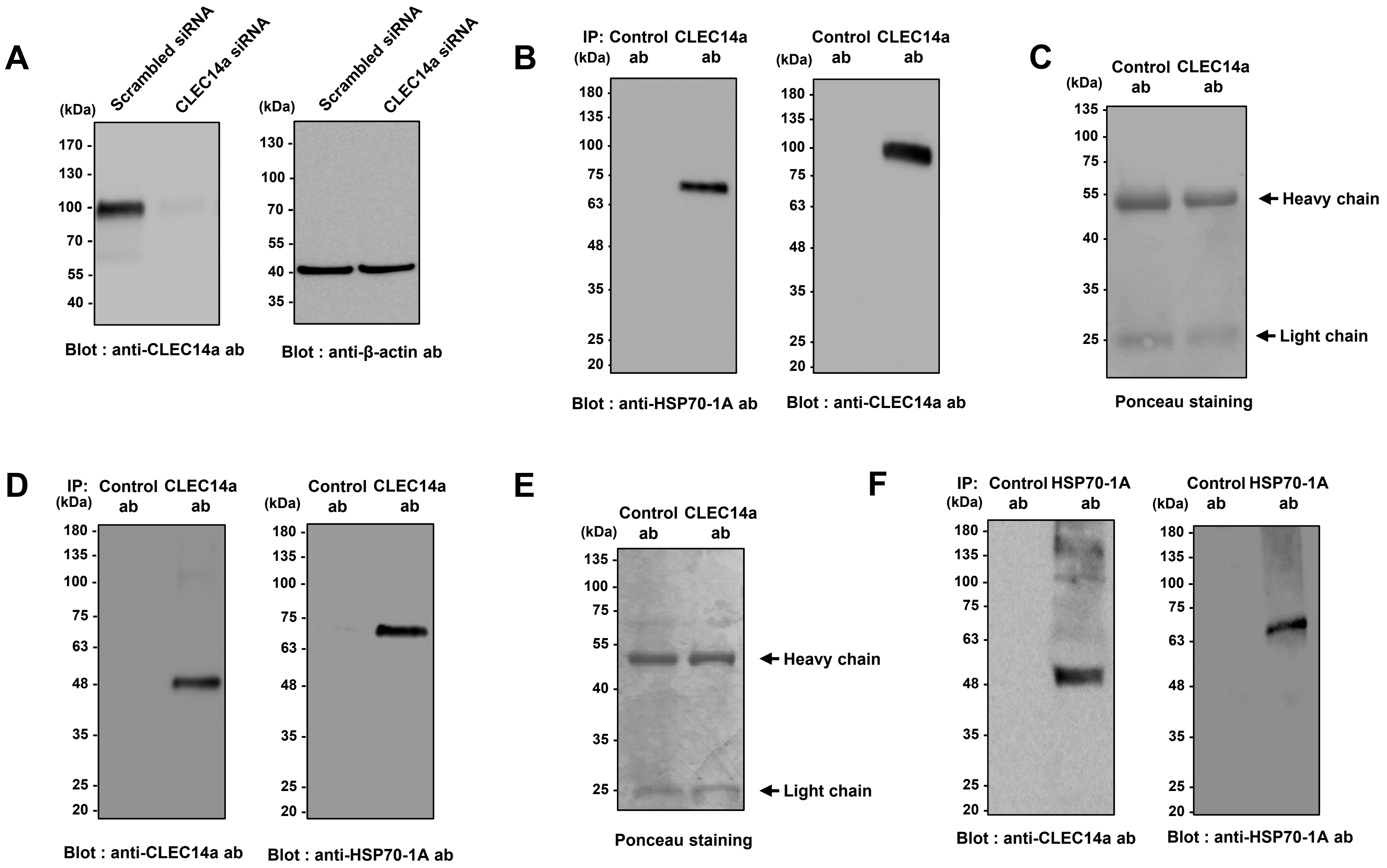
A
Scrambled siRNA
CLEC14a siRNA
(kDa)
170
130
100
70
55
40
Blot : anti-CLEC14a ab
(kDa)
130
100
70
55
40
35
Blot : anti-β-actin ab
B
IP: Control ab
CLEC14a ab
(kDa)
180
135
100
75
63
48
35
25
20
Blot : anti-HSP70-1A ab
Blot : anti-CLEC14a ab
C
Control ab
CLEC14a ab
Heavy chain
Light chain
Ponceau staining
D
IP: Control ab
CLEC14a ab
Blot : anti-CLEC14a ab
Blot : anti-HSP70-1A ab
E
Ponceau staining
F
IP: Control ab
HSP70-1A ab
Blot : anti-CLEC14a ab
Blot : anti-HSP70-1A ab

Fig. S7

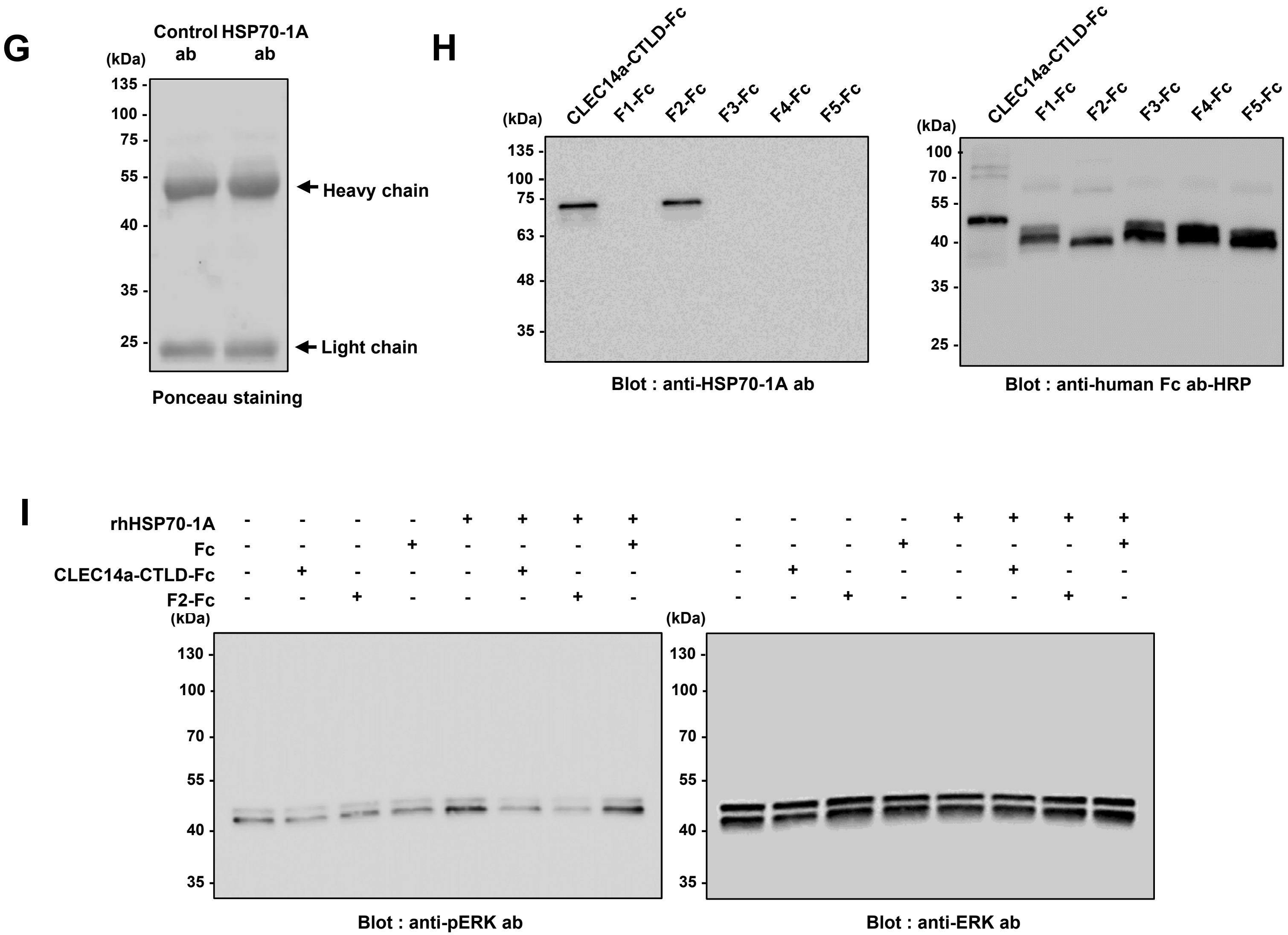

G
Control ab
HSP70-1A ab
(kDa)
135
100
75
55
40
35
25
Heavy chain
Light chain
Ponceau staining
H
CLEC14a-CTLD-Fc
F1-Fc
F2-Fc
F3-Fc
F4-Fc
F5-Fc
(kDa)
135
100
75
63
48
35
Blot : anti-HSP70-1A ab
(kDa)
100
70
55
40
35
25
Blot : anti-human Fc ab-HRP
I
rhHSP70-1A
Fc
CLEC14a-CTLD-Fc
F2-Fc
(kDa)
130
100
70
55
40
35
Blot : anti-pERK ab
Blot : anti-ERK ab

Fig. S7
